# Polypharmacy, multimorbidity, and socioeconomic inequalities in a Spanish region: a population-based cross-sectional study

**DOI:** 10.3389/fpubh.2026.1879178

**Published:** 2026-07-16

**Authors:** Gonzalo Aparicio-Rodriguez, Raúl Juárez-Vela, Ignacio M. Larráyoz, José Ángel Santos-Sánchez, Antonio Rodriguez-Calvo, Noelia Navas-Echazarreta, Urko Aguirre-Larracoechea, Vicente Gea-Caballero, Pilar Sánchez-Conde

**Affiliations:** 1 Interuniversity Doctoral Programme in Global Health, University of Zaragoza, University of La Rioja, Public University of Navarre, and University of Lleida, Logroño, Spain; 2 GRUPAC Research Group in Care, University of La Rioja, La Rioja, Spain; 3 Institute of Neurosciences of Castile and León (INCYL), University of Salamanca, and Biomedical Research Institute of Salamanca (IBSAL), Salamanca, Spain; 4 Biomarkers, Artificial Intelligence and Signaling (BIAS), University of La Rioja, La Rioja, Spain; 5 Faculty of Medicine, University of Salamanca, Salamanca, Spain; 6 Research Unit, Galdakao Hospital, Galdakao, Spain; 7 Faculty of Health Sciences, International University of Valencia, València, Spain

**Keywords:** aged, health status disparities, multimorbidity, polypharmacy, socioeconomic factors

## Abstract

**Background:**

Polypharmacy is closely linked to multimorbidity in older adults, but its distribution may also reflect social inequalities in health. We aimed to describe pharmacological burden and multimorbidity among individuals with polypharmacy in La Rioja, Spain, and to examine their relationship with age, sex, and socioeconomic conditions.

**Methods:**

This retrospective cross-sectional study included all individuals in La Rioja, Spain, who met the operational definition of polypharmacy between January 1 and December 31, 2024. Polypharmacy was defined as the dispensing of five or more distinct ATC4 medication groups, each dispensed at least six times during the study period. Only publicly funded prescriptions dispensed in community pharmacies were considered. Sociodemographic and clinical data were obtained from electronic health records. Pharmacological burden was quantified as the number of distinct ATC4 groups dispensed per individual and categorized as 5, 6–9, and ≥10 groups.

**Results:**

Among 33,534 individuals with polypharmacy, 54.7% were women and the mean age was 73.9 years. The mean pharmacological burden was 7.07 ATC4 groups (SD 2.17), with 58.4% of participants receiving 6–9 groups. Burden increased with age and was higher among individuals with chronic conditions, particularly congestive heart failure, renal disease, peripheral vascular disease, myocardial infarction, and chronic pulmonary disease. Sex differences were modest. In contrast, socioeconomic differences were marked: 71.8% of participants belonged to the low socioeconomic group, which also showed the highest proportion of individuals receiving ≥10 ATC4 groups (14.9%), compared with the middle (9.2%) and high (8.0%) socioeconomic groups. The highest burdens were observed in very low-income individuals and lower-income pensioners.

**Conclusions:**

In this population-based study, pharmacological burden among individuals with polypharmacy was concentrated on older adults, those with multimorbidity, and socioeconomically disadvantaged groups. These findings support interpreting polypharmacy not only as an indicator of clinical complexity, but also as a socially patterned health phenomenon. Efforts to optimize medication use should therefore integrate both clinical and socioeconomic vulnerability.

## 1 Introduction

Multimorbidity, commonly defined as the coexistence of two or more chronic conditions, is highly prevalent among older adults and represents a major challenge for healthcare systems because it is associated with poorer health status, lower quality of life, greater functional decline, and increased healthcare utilization (1). In this context, polypharmacy has emerged as one of the most relevant clinical consequences of multimorbidity. Although no single definition has been universally adopted, polypharmacy is most often described as the chronic use of five or more medications, whereas hyperpolypharmacy generally refers to the use of 10 or more (1, 2). As populations age and the prevalence of chronic disease continues to rise, the coexistence of multimorbidity and polypharmacy has become an increasingly frequent and clinically significant phenomenon (3, 4).

In Spain, the burden of polypharmacy among older adults is considerable. National data indicate that a substantial proportion of individuals aged 65 years and older use five or more chronic medications, with prevalence increasing markedly at older ages (5). This pattern reflects not only the accumulation of chronic conditions, but also the growing complexity of therapeutic management in later life. Two broad clinical profiles may be distinguished among individuals exposed to polypharmacy: those receiving multiple medications for a single complex condition, and those with multiple coexisting chronic diseases requiring several concurrent treatments (6). The latter pattern is the most common in routine clinical practice and is particularly relevant in aging populations.

Polypharmacy is not merely a numerical count of medications; it is a clinically meaningful marker of treatment complexity and vulnerability. A greater pharmacological burden has been associated with adverse drug reactions, drug–drug interactions, lower treatment adherence, functional impairment, geriatric syndromes, avoidable hospitalizations, and increased healthcare costs (7–9). For this reason, polypharmacy has become a major concern in clinical medicine, public health, and health services research, particularly in populations with chronic disease and frailty.

However, polypharmacy should not be understood exclusively as a consequence of biological aging or clinical morbidity. Increasing evidence suggests that medication burden is also shaped by the social determinants of health, that is, the conditions in which people are born, grow, work, live, and age, as well as the broader social and economic systems that structure daily life opportunities (10). Socioeconomic disadvantage is consistently associated with worse health outcomes, higher chronic disease burden, and shorter life expectancy (11, 12). These inequalities may also extend to patterns of medication use, as polypharmacy appears to be influenced not only by age and morbidity, but also by sex, income, educational opportunities, material deprivation, and differential access to health-promoting resources (6, 13, 14).

Despite this growing body of evidence, important gaps remain. Most studies on polypharmacy have focused either on older populations in general or on their association with multimorbidity, while fewer have examined how pharmacological burden is distributed within populations already exposed to polypharmacy according to socioeconomic position. In addition, evidence from population-based regional settings using real-world dispensing data remains limited, particularly in the Spanish context. Understanding whether pharmacological burden follows a social gradient is essential for identifying populations at increased clinical and social vulnerability and for informing strategies aimed at improving medication appropriateness and equity in care.

La Rioja, an autonomous community in northern Spain, offers an appropriate setting in which to explore these questions using healthcare and dispensing data at the population level. A previous study conducted in this region described patterns of medication use and their relationship with health determinants (15). Building on that work, a more specific analysis focused on polypharmacy and multimorbidity may provide further insight into how social inequalities are reflected in pharmacological burden in routine clinical practice.

Therefore, the aim of this study was to characterize pharmacological burden and multimorbidity among individuals with polypharmacy in La Rioja, Spain, and to examine their association with age, sex, and socioeconomic conditions.

## 2 Materials and methods

### 2.1 Study design

We conducted a retrospective cross-sectional observational study in La Rioja, an autonomous community in northern Spain, covering the period from January 1 to December 31, 2024.

### 2.2 Study setting and population

The study population included all individuals residing in La Rioja who met the operational definition of polypharmacy during the study period. Polypharmacy was defined as the dispensing of five or more distinct medications classified at the fourth level of the Anatomical Therapeutic Chemical (ATC4) system, with each ATC4 group dispensed six or more times between January 1 and December 31, 2024. This operational definition was selected to identify sustained pharmacological exposure over the study period and to reduce the inclusion of sporadic or short-term treatments. It should therefore be interpreted as a proxy measure of chronic pharmacological burden rather than as a direct measure of concurrent medication use.

Only prescriptions covered by the Spanish public healthcare system and dispensed in community pharmacies in La Rioja were included. Records with incomplete data in key study variables were excluded from analyses requiring those variables.

### 2.3 Data sources

Sociodemographic, clinical, and dispensing data were obtained from electronic health records and administrative healthcare databases. The available information included age, sex, postal code of residence, basic health zone, individual health card category, medications dispensed at the ATC4 and ATC5 levels, chronic conditions included in the multimorbidity profile, and tobacco and alcohol use.

### 2.4 Variables

The primary outcome was pharmacological burden, measured as the number of distinct ATC4 groups dispensed per individual during the study period. For descriptive analyses, pharmacological burden was examined both as a continuous variable and as a categorical variable grouped into three levels: 5 ATC4 groups, 6–9 ATC4 groups, and ≥10 ATC4 groups.

The main explanatory variables were age, sex, and socioeconomic status. Age was analyzed as a continuous variable. Sex was categorized as men or women according to the administrative health record. Socioeconomic status was assessed using the individual health card classification system Tarjeta Sanitaria Individual (TSI), which is based on income and employment or pension status and is used in the Spanish public healthcare system to determine pharmaceutical co-payment conditions. For analytical purposes, both the detailed TSI categories and a broader grouped socioeconomic classification were used: high, middle, low, and other.

Multimorbidity-related conditions were identified from the available clinical records and included myocardial infarction, congestive heart failure, peripheral vascular disease, cerebrovascular disease, dementia, chronic pulmonary disease, rheumatoid disease, peptic ulcer disease, mild liver disease, hemiplegia or paraplegia, renal disease, any cancer, metastatic cancer, and AIDS. Tobacco use and alcohol use were also collected when available.

### 2.5 Statistical analysis

Quantitative variables were summarized as mean and standard deviation (SD) or median and interquartile range (IQR), depending on their distribution. Categorical variables were described as frequencies and percentages.

Associations between categorical variables were assessed using the chi-square test. Because the distribution of the number of ATC4 groups was non-normal, comparisons between two groups were performed using the Mann–Whitney U-test, and comparisons across three or more groups were performed using the Kruskal–Wallis test. Contingency tables were used to summarize the distribution of pharmacological burden according to sex, socioeconomic status, and chronic conditions.

To address potential confounding, we fitted a multivariable logistic regression model with severe polypharmacy, defined as ≥10 distinct ATC4 medication groups, as the dependent variable. The model included age, sex, socioeconomic status, and Charlson comorbidity index as covariates. The Charlson index was categorized as 0, 1, 2, and ≥3. Model discrimination was assessed using the area under the receiver operating characteristic curve, and internal validation was performed using bootstrap optimism correction with 2,000 resamples.

All statistical analyses were performed using STATA/SE version 21.0 (StataCorp, College Station, TX, USA). A two-sided *p-value* < 0.05 was considered statistically significant.

### 2.6 Ethical considerations

This study routinely collected healthcare and dispensing data obtained from administrative and clinical records. All data were anonymized before analysis and processed in accordance with applicable data protection legislation and standards for biomedical research. The study protocol was reviewed and approved by the Research Ethics Committee for Medicines of La Rioja (Protocol No. PI 750). The research was conducted in accordance with local regulatory requirements and institutional policies. The requirement for written informed consent was waived by the ethics committee because the study was based exclusively on anonymized secondary data and involved no direct participant contact.

## 3 Results

### 3.1 Factors related to age and gender

A total of 33,534 individuals with polypharmacy were included in the study; 54.7% were women, and the mean age was 73.9 years. Women had a slightly higher mean number of ATC4 groups than men (7.10 vs. 7.04; *p* = 0.017). The 6–9 ATC4 category was the most common in both sexes, with no significant sex differences in the distribution of polypharmacy levels (*p* = 0.155). By contrast, age increased progressively across higher polypharmacy categories (*p* < 0.001). Among individuals with available socioeconomic status data (*n* = 33,514), significant differences were observed in both sex distribution and age across socioeconomic strata (*p* < 0.001), with a predominance of the low socioeconomic status group. Detailed sample characteristics are presented in Table 1.

**Table 1:** Sample characteristics and distribution of polypharmacy according to sex, age, and socioeconomic status.

Variable	Category/statistic	Total	Men	Women	*p*-value
Sex	*n* (%)	33,534	15,191 (45.3)	18,343 (54.7)	—
Age	Mean (SD)	73.93 (12.78)	—	—	—
Number of ATC4 groups	Mean (SD)	—	7.04 (2.17)	7.10 (2.18)	0.017
Polypharmacy categories	5 ATC4, *n* (%)	—	4,392 (28.9)	5,141 (28.0)	0.155
	6–9 ATC4, *n* (%)	—	8,823 (58.1)	10,738 (58.5)	
	≥10 ATC4, *n* (%)	—	1,976 (13.0)	2,464 (13.4)	
Age across polypharmacy categories	5 ATC4, mean (SD)	71.34 (13.27)	—	—	< 0.001
	6–9 ATC4, mean (SD)	74.60 (12.60)	—	—	
	≥10 ATC4, mean (SD)	76.53 (11.51)	—	—	
Socioeconomic status	Participants with available data, *n*	33,514	—	—	—
	High SES, *n* (%)	175	116 (66.3)	59 (33.7)	< 0.001
	Low SES, *n* (%)	24,067	9,806 (40.7)	14,261 (59.3)	
	Middle SES, *n* (%)	9,221	5,246 (56.9)	3,975 (43.1)	
	Other, *n* (%)	51	15 (29.4)	36 (70.6)	
Age across socioeconomic strata	High SES, mean (SD)	73.18 (10.07)	—	—	< 0.001
	Low SES, mean (SD)	74.41 (13.41)	—	—	
	Middle SES, mean (SD)	72.77 (10.89)	—	—	
	Other, mean (SD)	66.02 (13.40)	—	—	

SD, standard deviation; ATC4, fourth-level Anatomical Therapeutic Chemical subgroup; SES, socioeconomic status.

### 3.2 Conditions related to specific chronic diseases

The prevalence of chronic conditions varied across the study population. Renal disease was the most frequent condition (24.2%), followed by chronic pulmonary disease (19.2%), cerebrovascular disease (17.6%), congestive heart failure (16.4%), and any cancer (16.0%). In contrast, AIDS (0.3%), hemiplegia or paraplegia (1.4%), mild liver disease (2.3%), rheumatoid disease (2.6%), and metastatic cancer (3.0%) were uncommon.

Overall, the presence of chronic conditions was associated with a greater pharmacological burden, as reflected by a higher mean number of ATC4 groups. The largest differences were observed for congestive heart failure (8.18 vs. 6.85), renal disease (7.74 vs. 6.86), peripheral vascular disease (7.84 vs. 6.99), myocardial infarction (7.72 vs. 7.00), and chronic pulmonary disease (7.62 vs. 6.94). Median ATC4 values showed a similar pattern.

Most comparisons were statistically significant. However, no significant differences in ATC4 burden were observed for metastatic cancer (*p* = 0.063) or AIDS (*p* = 0.258). Detailed results are shown in Table 2.

**Table 2:** Prevalence of chronic conditions and ATC4 burden according to disease status.

Condition	No, *n* (%)	Yes, *n* (%)	No, mean ATC4 (SD)	Yes, mean ATC4 (SD)	No, median (P25–P75)	Yes, median (P25–P75)	*p*-value
Myocardial infarction	30,434 (90.8)	3,099 (9.2)	7.00 (2.13)	7.72 (2.47)	6.00 (5.00–8.00)	7.00 (6.00–9.00)	< 0.001
Congestive heart failure	28,048 (83.6)	5,485 (16.4)	6.85 (2.03)	8.18 (2.51)	6.00 (5.00–8.00)	8.00 (6.00–10.00)	< 0.001
Peripheral vascular disease	30,441 (90.8)	3,092 (9.2)	6.99 (2.12)	7.84 (2.55)	6.00 (5.00–8.00)	7.00 (6.00–9.00)	< 0.001
Cerebrovascular disease	27,622 (82.4)	5,911 (17.6)	6.96 (2.11)	7.57 (2.38)	6.00 (5.00–8.00)	7.00 (6.00–9.00)	< 0.001
Dementia	31,384 (93.6)	2,149 (6.4)	7.05 (2.17)	7.43 (2.23)	6.00 (5.00–8.00)	7.00 (6.00–9.00)	< 0.001
Chronic pulmonary disease	27,111 (80.8)	6,422 (19.2)	6.94 (2.06)	7.62 (2.54)	6.00 (5.00–8.00)	7.00 (6.00–9.00)	< 0.001
Rheumatoid disease	32,669 (97.4)	864 (2.6)	7.06 (2.17)	7.32 (2.29)	6.00 (5.00–8.00)	7.00 (6.00–8.25)	< 0.001
Peptic ulcer disease	32,146 (95.9)	1,387 (4.1)	7.06 (2.17)	7.23 (2.22)	6.00 (5.00–8.00)	7.00 (5.00–8.00)	0.004
Mild liver disease	32,775 (97.7)	758 (2.3)	7.06 (2.17)	7.47 (2.51)	6.00 (5.00–8.00)	7.00 (5.25–9.00)	< 0.001
Hemiplegia or paraplegia	33,058 (98.6)	475 (1.4)	7.06 (2.17)	7.74 (2.40)	6.00 (5.00–8.00)	7.00 (6.00–9.00)	< 0.001
Renal disease	25,432 (75.8)	8,101 (24.2)	6.86 (2.04)	7.74 (2.43)	6.00 (5.00–8.00)	7.00 (6.00–9.00)	< 0.001
Any cancer	28,183 (84.0)	5,350 (16.0)	7.05 (2.16)	7.18 (2.23)	6.00 (5.00–8.00)	7.00 (5.00–8.00)	< 0.001
Metastatic cancer	32,541 (97.0)	992 (3.0)	7.07 (2.17)	7.25 (2.34)	6.00 (5.00–8.00)	7.00 (5.00–8.00)	0.063
AIDS	33,435 (99.7)	98 (0.3)	7.07 (2.17)	7.02 (2.53)	6.00 (5.00–8.00)	6.00 (5.00–7.75)	0.258

SD, standard deviation; P25–P75, 25th−75th percentile; ATC4, fourth-level Anatomical Therapeutic Chemical subgroup.

**Figure 1:**
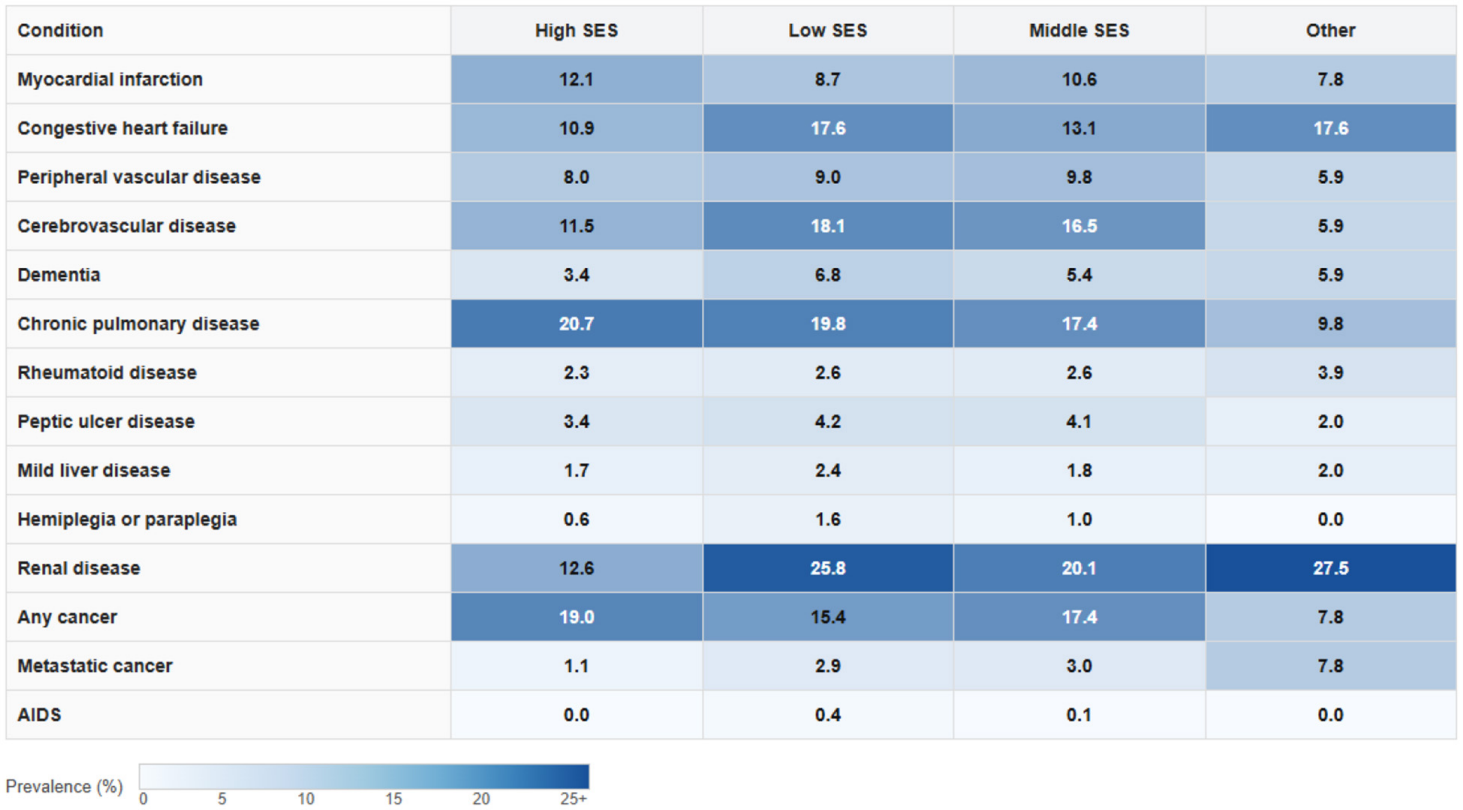
Heatmap of the prevalence of chronic conditions according to socioeconomic. Color intensity represents the percentage of individuals with each chronic condition within each socioeconomic stratum. Percentages correspond to the proportion of participants with the condition (“yes”) in each category. SES, socioeconomic status.

The heatmap in Figure 1 highlights differences in the distribution of chronic conditions across socioeconomic strata. A clearer burden in the low-SES group was observed for renal disease, cerebrovascular disease, congestive heart failure, and dementia, whereas myocardial infarction and any cancer showed relatively higher proportions in the high-SES and middle-SES groups. Although several associations reached statistical significance, the magnitude of differences varied across conditions and was modest for some of them. Conditions such as peripheral vascular disease, rheumatoid disease, peptic ulcer disease, and metastatic cancer showed little variation across socioeconomic categories.

### 3.3 Socioeconomic level

The socioeconomic profile of the study population was markedly skewed toward disadvantaged groups. Under the detailed TSI classification, very low-income individuals constituted the largest subgroup (35.5%), followed by pensioners with income < €18,000 (28.0%) and pensioners with income €18,000–€100,000 (22.9%). Consistently, in the broader socioeconomic classification, 71.8% of participants were categorized as low socioeconomic level, whereas only 0.5% were classified as high socioeconomic level.

A significant gradient in pharmacological burden was observed across TSI categories (*p* < 0.001). The highest ATC4 burden was found among individuals in the very low-income group and among lower-income pensioners, while the lowest values were observed in the active population groups and among those with higher income levels. This pattern suggests that socioeconomic disadvantage is associated with a greater complexity of pharmacological treatment. See Table 3.

**Table 3:** Distribution of participants according to socioeconomic classification and pharmacological burden.

Variable	Category	*n* (%)	Mean number of ATC4 groups (SD)	Median (P25–P75)	*p*-value
Detailed TSI category	Active population, income < €18,000	2,771 (8.3)	6.23 (1.52)	6.00 (5.00–7.00)	< 0.001
	Active population, income €18,000–€100,000	1,546 (4.6)	6.12 (1.49)	6.00 (5.00–7.00)	< 0.001
	Unknown	20 (0.1)	6.85 (1.79)	6.50 (5.75–8.00)	< 0.001
	Very low income	11,907 (35.5)	7.38 (2.34)	7.00 (6.00–9.00)	< 0.001
	Non-resident/foreigner	50 (0.1)	6.12 (1.38)	6.00 (5.00–7.00)	< 0.001
	Pensioner income < €18,000	9,389 (28.0)	7.24 (2.24)	7.00 (5.00–8.00)	< 0.001
	Pensioner, income €18,000–€100,000	7,675 (22.9)	6.89 (1.99)	6.00 (5.00–8.00)	< 0.001
	Income >€100,000	175 (0.5)	6.55 (1.79)	6.00 (5.00–7.00)	< 0.001
	Uninsured	1 (0.0)	5.00 (NA)	5.00 (5.00–5.00)	< 0.001
Socioeconomic level	High	175 (0.5)	6.55 (1.79)	6.00 (5.00–7.00)	< 0.001
	Low	24,067 (71.8)	7.19 (2.25)	7.00 (5.00–8.00)	< 0.001
	Middle	9,221 (27.5)	6.76 (1.94)	6.00 (5.00–8.00)	< 0.001
	Other	51 (0.2)	6.10 (1.37)	6.00 (5.00–7.00)	< 0.001

**Figure 2:**
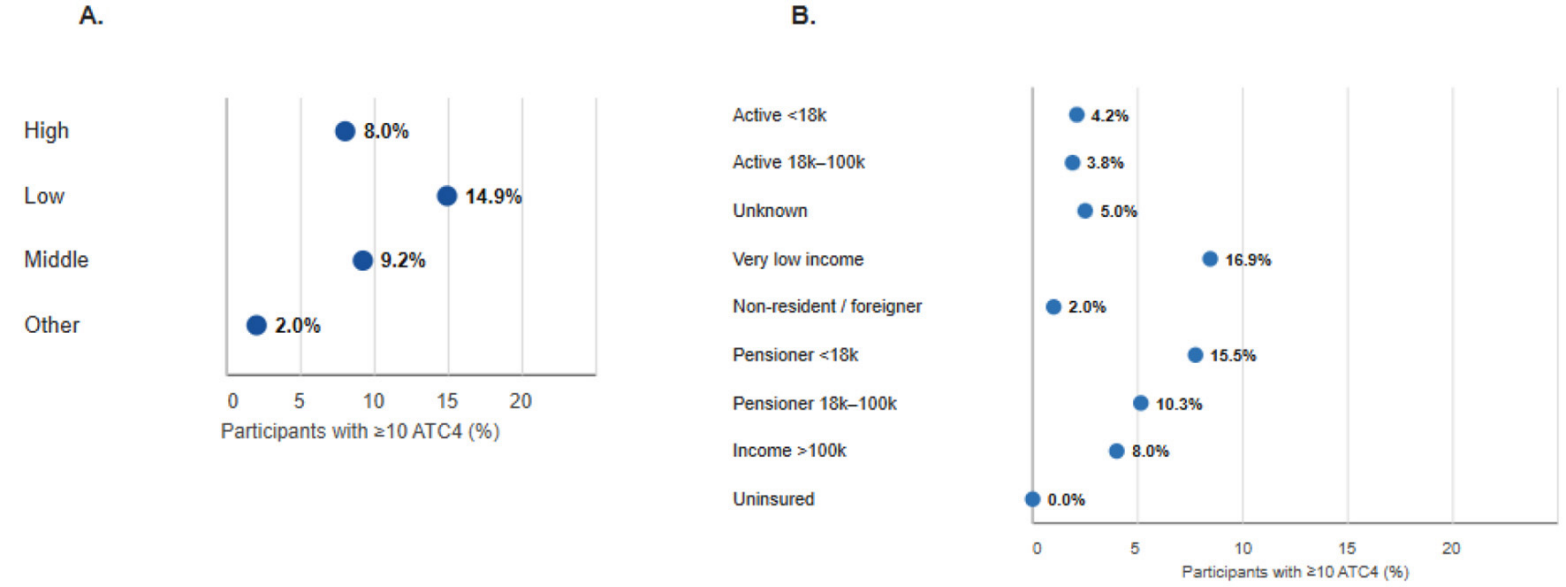
**(A)** Socioeconomic level. **(B)** Detailed TSI category. Proportion of participants receiving ≥10 ATC4 groups according to socioeconomic level and detailed TSI category. TSI status was categorized as follows: uninsured (NOFAR); exempt/very low income or social exclusion (TSI 001); pensioners with annual income < €18,000 (TSI 002-01) or €18,000–€100,000 (TSI 002-02); employed/active individuals with annual income < €18,000 (Low-TSI 003) or €18,000–€100,000 (Middle-TSI 004); annual income ≥€100,000 (High-TSI 005); mutual insurance beneficiaries (TSI 006); non-La Rioja residents or foreign patients (F003/F004).

Figure 2A shows the broader socioeconomic classification and Figure 2B shows the detailed TSI categories. Values indicate the percentage of participants in each category receiving ≥10 ATC4 groups and the proportion of participants receiving ≥10 ATC4 groups showed a clear socioeconomic gradient. At the broader socioeconomic level, this proportion was highest in the low socioeconomic group (14.9%), followed by the middle (9.2%) and high (8.0%) groups, whereas the other category showed the lowest proportion (2.0%). A similar pattern was observed across detailed TSI categories, with the highest proportions of intensive pharmacological burden found in the very low-income group (16.9%) and in pensioners with income < €18,000 (15.5%). By contrast, the corresponding proportions were substantially lower in the active population categories (4.2% and 3.8%). Overall, these findings support a greater concentration of high pharmacological burden among socioeconomically disadvantaged groups.

### 3.4 Number of ATC4 codes

**Figure 3:**
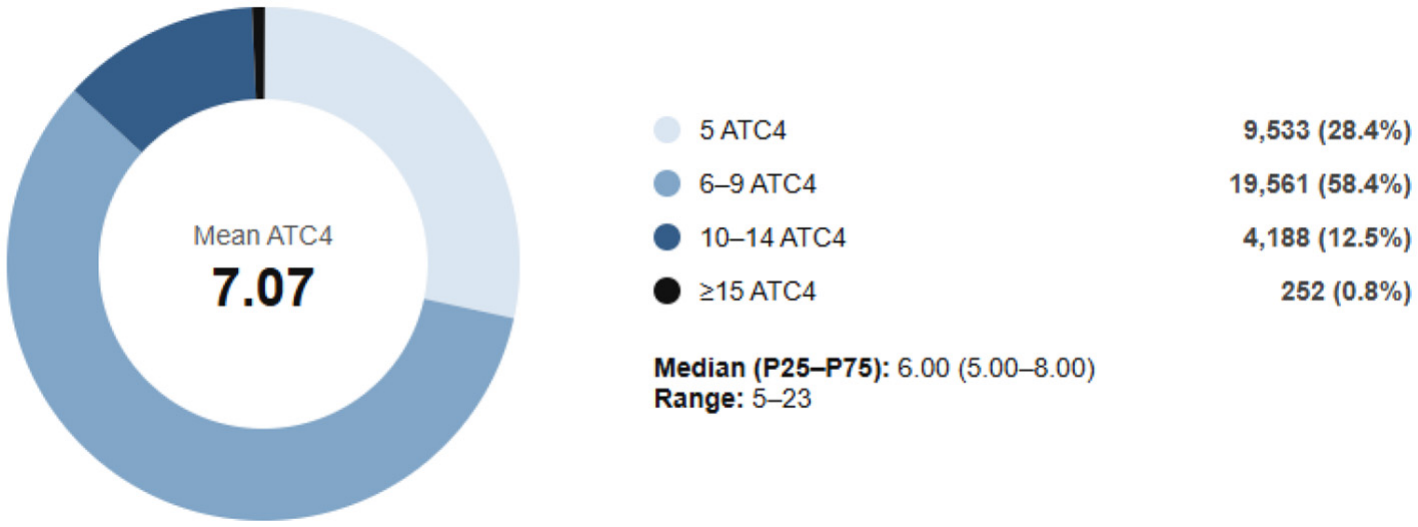
Distribution of pharmacological burden according to the number of ATC4 groups. The donut chart shows grouped categories of ATC4 burden for visualization purposes: 5, 6–9, 10–14, and ≥15 ATC4 groups. The central value indicates the mean number of ATC4 groups in the study population.

The distribution of pharmacological burden was right skewed toward lower numbers of ATC4 groups, although a substantial proportion of participants showed moderate medication complexity. The most frequent category was 5 ATC4 groups (28.4%), followed by 6 (22.1%) and 7 (16.4%) groups. When categories were collapsed for visualization, 58.4% of participants were receiving 6–9 ATC4 groups, whereas 12.5% were receiving 10–14 groups and fewer than 1% were receiving 15 or more groups. Overall, the mean number of ATC4 groups was 7.07 (SD 2.17), with a median of 6.00 (P25–P75: 5.00–8.00), indicating that most participants clustered around the lower end of the inclusion range but with a progressively decreasing tail toward higher pharmacological burden. See Table 4 and Figure 3.

**Table 4:** Distribution of pharmacological burden according to the number of ATC4 groups.

Number of ATC4 groups	*n* (%)
5	9,533 (28.4)
6	7,397 (22.1)
7	5,514 (16.4)
8	3,965 (11.8)
9	2,685 (8.0)
10	1,761 (5.3)
11	1,097 (3.3)
12	718 (2.1)
13	362 (1.1)
14	250 (0.7)
15	116 (0.3)
16	64 (0.2)
17	35 (0.1)
18	17 (0.1)
19–23	20 (0.1)
Mean (SD)	7.07 (2.17)
Median (P25–P75)	6.00 (5.00–8.00)
Min–max	5.00–23.00

### 3.5 Models of multivariable logistic regression

We performed the multivariable logistic regression model as well, severe polypharmacy, defined as the dispensing of ≥10 distinct ATC4 medication groups, was independently associated with older age, higher comorbidity burden, and socioeconomic status. After adjustment for age, sex, Charlson comorbidity index, and socioeconomic status, individuals with a Charlson index ≥3 had markedly higher odds of severe polypharmacy compared with those with a Charlson index of 0 (adjusted OR 3.15; 95% CI 2.89–3.43; *p* < 0.001). Middle and high socioeconomic status were associated with lower odds of severe polypharmacy compared with low socioeconomic status, with adjusted ORs of 0.62 (95% CI 0.57–0.67; *p* < 0.001) and 0.56 (95% CI 0.32–0.98; *p* = 0.041), respectively. Sex was not independently associated with severe polypharmacy. The model showed modest discrimination, with an apparent AUC of 0.6528 and an optimism-corrected AUC of 0.6520 after 2,000 bootstrap resamples. See Tables 5, 6.

**Table 5:** Multivariable logistic regression model for severe polypharmacy, defined as ≥10 ATC4 medication groups.

Variable	Category/unit	Adjusted OR	95% CI	*p*-value
Age	Per 1-year increase	1.01	1.00–1.01	< 0.001
Sex	Men	Reference	—	—
	Women	1.02	0.96–1.09	0.512
Charlson comorbidity index	0	Reference	—	—
	1	1.74	1.58–1.92	< 0.001
	2	1.53	1.38–1.69	< 0.001
	≥3	3.15	2.89–3.43	< 0.001
Socioeconomic status	Low	Reference	—	—
	Middle	0.62	0.57–0.67	< 0.001
	High	0.56	0.32–0.98	0.041
	Other	0.12	0.02–0.88	0.037

**Table 6:** Model performance.

Model performance metric	Value
Number of observations	33,513
Number of events, ≥10 ATC4 groups	4,439
AUC apparent	0.6528
Optimism-corrected AUC, bootstrap B = 2,000	0.6520
95% CI for optimism-corrected AUC	0.6511–0.6530
Brier score	0.1110
Calibration intercept	0.0000
Calibration slope	1.0000

Multivariable logistic regression model with severe polypharmacy, defined as the dispensing of ≥10 distinct ATC4 medication groups, as the dependent variable. The model was adjusted simultaneously for age, sex, Charlson comorbidity index, and socioeconomic status. The Charlson comorbidity index was used as a summary measure of clinical morbidity. The discriminatory performance of the model was assessed using the area under the receiver operating characteristic curve. Internal validation was performed using bootstrap optimism correction with 2,000 resamples.

OR, odds ratio; CI, confidence interval; AUC, area under the curve; ATC4, fourth-level Anatomical Therapeutic Chemical subgroup.

**Figure 4:**
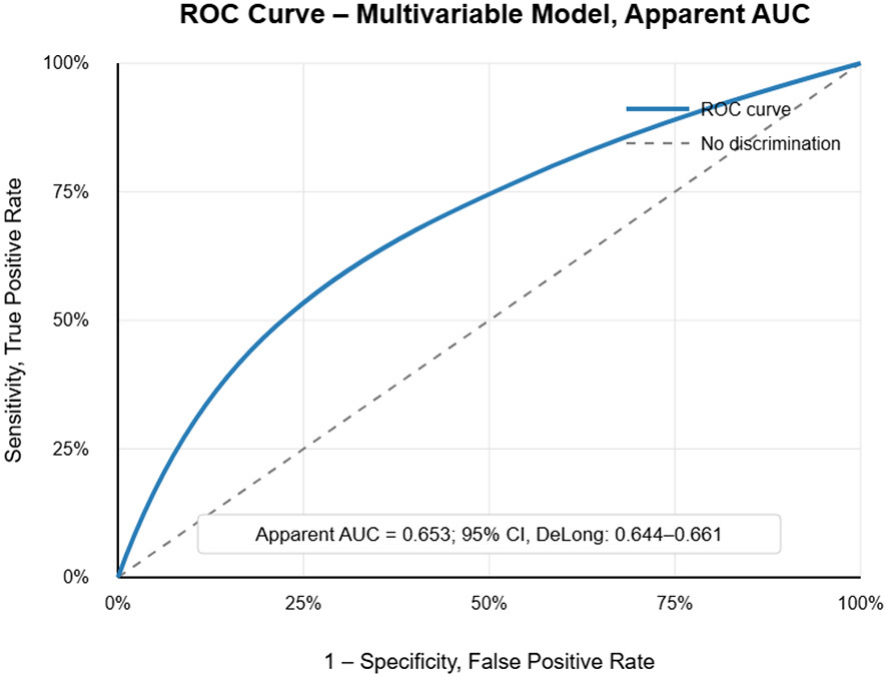
Receiver operating characteristic curve.

Model discrimination was assessed using the receiver operating characteristic curve. The model showed modest discriminatory ability, with an apparent AUC of 0.6528 and an optimism-corrected AUC of 0.6520 after 2,000 bootstrap resamples. See Figure 4.

## 4 Discussion

The present study provides a population-based characterization of individuals with polypharmacy in La Rioja and shows that pharmacological burden is closely linked not only to age and multimorbidity, but also to socioeconomic disadvantage. Although the overall prevalence of polypharmacy was consistent with national estimates, the distribution of therapeutic burden was not socially neutral. Greater pharmacological burden was concentrated among older individuals, those with chronic comorbidity, and socially disadvantaged groups. Sex differences were comparatively modest in terms of treatment intensity, although women represented a slightly higher proportion of the population with polypharmacy.

In 2024, 33,534 individuals in La Rioja met the operational definition of polypharmacy, representing 10.3% of the total population. This figure is consistent with national estimates reported by the Spanish Ministry of Health (16). The demographic profile of the cohort was also broadly aligned with previous reports, with women accounting for 54.7% of individuals with polypharmacy (17–19). This pattern may reflect women's longer life expectancy, a higher prevalence of chronic non-fatal conditions, and greater use of healthcare services. In addition, the higher proportion of women in lower socioeconomic strata is consistent with previous findings from the EpiChron cohort, which linked these inequalities to gendered differences in social and living conditions (20). Although women showed a slightly higher mean pharmacological burden than men, the magnitude of this difference was small, suggesting that sex-related differences may be more apparent in prevalence than in treatment intensity.

The mean age of the cohort was 73.9 years, which is very close to that reported in national data from the Ministry of Health (16). Previous studies have reported somewhat different mean ages, such as 77.3 years (18) and 69.7 years (19), although such variation is likely explained by differences in inclusion criteria and study populations. The positive association between age and pharmacological burden observed in our study is consistent with prior research linking aging, multimorbidity, and therapeutic complexity (16, 21–24). Older adults are more likely to accumulate chronic conditions requiring long-term treatment, thereby increasing sustained exposure to multiple pharmacological groups.

Regarding treatment intensity, the mean pharmacological burden was 7.07 ATC4 groups. Although this figure is slightly lower than that reported in some previous studies, such as the estimate of 8 medications described elsewhere (25), such differences should be interpreted in light of varying operational definitions of polypharmacy. In our study, no substantial sex differences were observed in pharmacological burden, which is consistent with national findings showing similar medication counts in men and women despite some differences in prevalence (16).

Multimorbidity was a defining feature of the population studied. Kidney disease, chronic pulmonary disease, cerebrovascular disease, and heart failure were among the most prevalent conditions, a pattern that is broadly consistent with reports from the Ministry of Health and the World Health Organization (17, 26). Other studies have additionally highlighted conditions such as dyslipidemia, osteoarthritis, hypertension, or musculoskeletal disorders (27, 28), although these differences are likely related to the specific morbidity lists used in each investigation. In our analysis, heart failure, renal disease, peripheral vascular disease, myocardial infarction, and chronic pulmonary disease were the conditions most strongly associated with greater pharmacological burden. This finding is clinically plausible, given that these disorders frequently coexist and often require complex long-term treatment regimens, contributing substantially to therapeutic burden (29, 30).

Our results also underscore the extent to which pharmacological burden is embedded within a broader social and clinical context. In the multivariable logistic regression model, severe polypharmacy remained independently associated with older age, higher Charlson comorbidity burden, and lower socioeconomic status. The model showed modest discriminatory ability, with an apparent AUC of 0.653, indicating that severe polypharmacy is likely driven by multiple interrelated factors rather than by clinical morbidity alone. In line with this finding, conditions such as cerebrovascular disease, renal disease, dementia, and heart failure were more frequent among individuals with lower socioeconomic status. This is consistent with previous evidence showing that socioeconomic disadvantage is associated with poorer health outcomes, greater chronic disease burden, and fewer opportunities to adopt and sustain healthy lifestyles (31, 32). These pathways may promote the earlier onset and accumulation of multimorbidity, thereby contributing to a greater need for pharmacological treatment in socially disadvantaged populations.

One of the main findings of this study is the clear socioeconomic gradient in polypharmacy. Individuals in lower socioeconomic groups accounted for most cases, and the highest treatment burden categories were also disproportionately concentrated in these groups. According to the TSI classification, the very low-income group (TSI001) represented 35.5% of all individuals with polypharmacy, followed by lower-income pensioners (TSI002-01), who accounted for 28.0%. These findings are consistent with national data from the Ministry of Health (16) and with previous literature linking polypharmacy to lower income and socioeconomic disadvantage (33). They reinforce the interpretation that socioeconomic position is not merely a contextual factor, but an important correlate of pharmacological burden.

The role of the pharmaceutical co-payment system should also be considered. In Spain, lower-income groups and some pensioners are subject to minimal or reduced out-of-pocket costs, which may facilitate access to prescribed medications. This may partly influence dispensing patterns, as suggested in studies such as the TILDA cohort (34). However, reduced co-payment alone is unlikely to explain the magnitude of the observed differences. More plausibly, the greater pharmacological burden among disadvantaged groups reflects the combined association of higher multimorbidity, earlier disease onset, and greater clinical complexity. In this regard, our findings are consistent with broader evidence showing that chronic disease burden, frequent healthcare use, and hospitalization are socially patterned and disproportionately concentrated in more deprived populations (35, 36).

## 5 Clinical implications

These findings have relevant implications for both clinical practice and public health. First, they suggest that polypharmacy should not be approached solely as a prescribing issue, but also as a manifestation of cumulative clinical and social vulnerability. Medication review strategies should therefore move beyond the simple counting of drugs and incorporate the broader context in which treatment is prescribed, managed, and sustained.

Second, the concentration of greater pharmacological burden in socioeconomically disadvantaged groups indicates that interventions to improve medication appropriateness should be tailored to patients at increased social risk. In these populations, treatment adherence, health literacy, competing social needs, and barriers to self-management may influence the effectiveness and safety of pharmacological care. Integrating socioeconomic information into medication review and deprescribing strategies may therefore improve both equity and clinical outcomes.

Third, these results support strengthening the role of multidisciplinary medication management, particularly through closer collaboration between primary care, hospital care, and community pharmacy. Structured medication review programs may be especially valuable in patients with multimorbidity, high therapeutic burden, and social disadvantage, where the risk of inappropriate prescribing, adverse drug events, and avoidable healthcare use is likely to be greater.

At the policy level, the findings also highlight the importance of incorporating social determinants of health into chronic care and pharmaceutical care strategies. Approaches aimed at optimizing medication use should consider not only disease burden, but also the unequal social distribution of that burden across the population.

From an implementation perspective, these findings support the integration of automated risk-stratification tools into electronic health records to combine clinical, pharmacological, and socioeconomic information and identify polymedicated patients at higher risk of severe therapeutic burden. Collaborative medication review pathways involving physicians and pharmacists could further facilitate deprescribing strategies tailored to patients' clinical complexity and socioeconomic circumstances. At the healthcare management level, these results may contribute to moving beyond simple medication counts toward comprehensive treatment optimization strategies that prioritize socially vulnerable patients with greater multimorbidity.

## 6 Limitations

Several limitations should be considered when interpreting these findings. First, the retrospective cross-sectional design limits causal inference and does not allow the temporal relationship between socioeconomic conditions, multimorbidity, and pharmacological burden to be established. Therefore, the associations observed should be interpreted as descriptive and hypothesis-generating.

Second, medication exposure was assessed using dispensing records. These data provide a reliable proxy for prescribed and supplied treatments in routine care, particularly for chronic medication use, but they do not necessarily confirm actual medication intake or adherence. Thus, pharmacological burden should be interpreted as dispensed treatment burden rather than confirmed consumed medication.

Third, pharmacological burden was defined according to repeated dispensing of distinct ATC4 groups. This approach enables a standardized and reproducible assessment of exposure to different therapeutic groups, but it may not fully capture simultaneous medication use, dosage, treatment duration, or clinical appropriateness at the individual level.

Fourth, the analysis included prescriptions covered by the public healthcare system and dispensed through community pharmacies in La Rioja. Consequently, over-the-counter medications, private prescriptions, and medicines dispensed exclusively in hospital settings were not included. However, given the near-universal coverage of the regional public healthcare system and the subsidized access to chronic treatments, the dataset is expected to capture most long-term pharmacological exposure in this population.

Finally, some variables recorded in routine clinical practice, particularly lifestyle-related factors such as tobacco and alcohol use, may be subject to incomplete or infrequently updated registration. For this reason, these variables were not included in the final models. Their exclusion should be considered when interpreting the results, although the main analyses included key demographic, socioeconomic, and clinical variables.

Despite these considerations, the study provides population-level evidence based on real-world clinical and dispensing data and offers relevant insight into the social patterning of polypharmacy in a regional Spanish population. Its strengths include the comprehensive population-based design and the integration of clinical, pharmacological, and socioeconomic information.

## 7 Conclusions

This study shows that polypharmacy in La Rioja is strongly associated with older age, multimorbidity, and socioeconomic disadvantages. Pharmacological burden was especially concentrated in lower-income groups, indicating that polypharmacy should be understood not only as a marker of clinical complexity, but also as a socially patterned health phenomenon.

These findings support the need to move beyond a purely biomedical model in the management of polypharmacy. Strategies to optimize medication use should incorporate social vulnerability, alongside clinical complexity, in order to promote more equitable, safer, and more efficient care.

## Data Availability

The raw data supporting the conclusions of this article will be made available by the authors, without undue reservation.
